# Chronic Myeloid Leukemia with e19a2 *BCR-ABL1* Transcripts and Marked Thrombocytosis: The Role of Molecular Monitoring

**DOI:** 10.1155/2012/458716

**Published:** 2012-07-02

**Authors:** Stephen E. Langabeer, Sarah L. McCarron, Johanna Kelly, Janusz Krawczyk, Suzanne McPherson, Kanthi Perera, Philip T. Murphy

**Affiliations:** ^1^Cancer Molecular Diagnostics, St. James's Hospital, Dublin 8, Ireland; ^2^National Centre for Medical Genetics, Our Lady's Children's Hospital, Dublin 12, Ireland; ^3^Department of Haematology, Midland Regional Hospital, Tullamore, Ireland; ^4^Department of Haematology, Beaumont Hospital, Dublin 9, Ireland

## Abstract

While most patients with chronic myeloid leukemia (CML) express either e13a2 or e14a2 *BCR-ABL1* transcripts, a significant minority expresses variant transcripts, of which e19a2 is the most common. Although considered to have a relatively favourable outcome, reported responses to tyrosine kinase inhibitor (TKI) therapy are variable with molecular monitoring in CML patients with e19a2 *BCR-ABL1* transcripts rarely reported. A case of e19a2 *BCR-ABL1* CML with marked thrombocytosis is described in which the value of molecular monitoring is emphasised during treatment interruptions, dose reductions, and changes. This case serves to demonstrate the requirement for prospective real-time quantitative PCR (RQ-PCR) assays for patients with variant *BCR-ABL1* transcript types and standardisation of such assays to enable modern patient management.

## 1. Introduction

The *BCR-ABL1* fusion gene is the molecular hallmark and causative event of CML. Most CML patients express either e13a2 or e14a2 *BCR-ABL1* fusion transcripts but approximately 5% of patients express variant transcripts that may involve fusion of alternative exons, insertions, or breakpoints within exons. Of these variant *BCR-ABL1* fusions, the e19a2 is the most common with approximately 50 cases reported to date. CML with e19a2 *BCR-ABL1*, that encodes a 230 kDa protein [1], was initially reported in neutrophilic CML with a relatively indolent clinical course [2] but has subsequently been reported in typical CML presenting in all phases [3]. While TKI therapy is considered the optimal frontline treatment for CML with quantitative molecular responses predictive of overall and progression-free survival [4], review of the literature indicates that hematological and cytogenetic responses to TKIs are variable in e19a2 *BCR-ABL1* CML [3, 5–11], although this may be biased by reporting of cases with atypical features. Molecular responses have rarely been reported with real-time quantitative polymerase chain reaction (RQ-PCR) monitoring thus far documented in only two patients [12, 13], suggesting that this important element of patient management may be forsaken in a significant proportion of patients that express this and other variant transcripts types. Although an elevated platelet count is observed at presentation in a number of CML patients, marked thrombocytosis (platelets > 1000 × 10^9^/L) is uncommon and often associated with the e19a2 *BCR-ABL1* transcript type [14]. The diagnosis, course, and molecular monitoring of a patient with e19a2 *BCR-ABL1* CML who presented with a marked thrombocytosis is described.

## 2. Case Report

An eighty-year-old male with a history of Duke's C colon carcinoma in remission presented with an abnormal full blood count: Hb 9.9 g/dL, PLT 1401 × 10^9^/L, WBC 31.7 × 10^9^/L which comprised neutrophils 25.2 × 10^9^/L, lymphocytes 2.1 × 10^9^/L, monocytes 0.94 × 10^9^/L, eosinophils 1.75 × 10^9^/L, basophils 0.85 × 10^9^/L, and blasts 0.1 × 10^9^/L. The blood film showed thrombocytosis, marked platelet anisocytosis, neutrophilia with left shift, and rouleaux with the patient having an elevated lactate dehydrogenase of 554 IU/L. The bone marrow aspirate was hypercellular with particles present and showed significant myeloid hyperplasia and plentiful megakaryocytes, often present in groups with polymorphic morphology including hypolobulated and mononuclear forms. Cytogenetic studies were not performed but a standard reverse-transcriptase polymerase chain reaction methodology for detection of *BCR-ABL1* transcripts [15] showed a single band that was not consistent with the common e13a2 or e14a2 *BCR-ABL1* transcripts. Direct sequencing demonstrated the presence of an e19a2 *BCR-ABL1* fusion with a final diagnosis of chronic phase CML with a high-risk Sokal score of 3.15.

**Figure 1:**
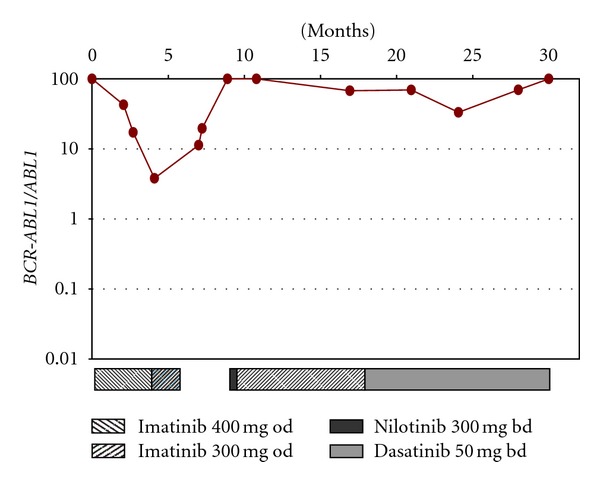
RQ-PCR monitoring of e19a2 *BCR-ABL1 *transcripts.

The patient commenced on imatinib 400 mg od with aspirin 75 mg once daily. After three weeks of therapy, when the platelet count was still greater than 1000 × 10^9^/L, a deep vein thrombosis (DVT) of the left popliteal vein extending into the common femoral vein was diagnosed prompting treatment with low molecular weight heparin. Complete cytogenetic remission was achieved (0/50 Ph+ metaphases) after three months of imatinib therapy with RQ-PCR for e19a2 *BCR-ABL1* transcripts performed as previously described [13] demonstrating an initial decrease in *BCR-ABL1* transcript level (*BCR-ABL1/ABL1* 3.8%, Figure 1). Over the following months the patient complained of intermittent abdominal pain and diarrhoea and because of these symptoms, imatinib was initially reduced to 300 mg od with several further treatment interruptions due to the abdominal symptoms and recurrent rash. The patient subsequently underwent gastroenterology investigations and was diagnosed with intermittent small bowel obstruction secondary to adhesions. He underwent a laparotomy but perioperatively had recurrence of DVT and was again fully anticoagulated. As gastrointestinal symptoms persisted after surgery, imatinib was stopped reflected by a rise in *BCR-ABL1* transcripts to high levels (Figure 1). Treatment with nilotinib 300 mg bd was instigated but halted after three weeks due to recurrence of both diarrhoea and a rash. Nine months from initial diagnosis the patient was restarted with the best tolerated therapy of imatinib 300 mg od. He continued to have significant symptoms including intermittent diarrhoea and abdominal pains with adherence questionable. The patient was switched to dasatinib 50 mg twice daily 18 months after diagnosis achieving a partial cytogenetic remission at 21 and 24 months (6/50 Ph+ and 12/50 Ph+, resp.) with only a slight molecular response (Figure 1). He remains in hematological remission with relatively improved gastrointestinal symptoms and adherence but at 30 months has worsening cytogenetic and molecular responses: 42/50 Ph+ and clonal evolution in the form of an extra der(22) or an isochromosome of the der(22) and *BCR-ABL1/ABL1* 100%.

## 3. Discussion

Thrombocytosis is a relatively common presenting feature of CML but platelet counts >1000 × 10^9^/L are rare. An association with e19a2 *BCR-ABL1* transcripts in CML patients and marked thrombocytosis has previously been documented [14], corroborated by the characterisation of a distinctive high platelet count in a transgenic mouse model expressing p230 BCR-ABL1 [16], and supported by the findings in the patient described herein. As to whether the significant thrombocytosis contributed to the DVT in this patient remains unknown: a prompt targeted reduction in platelets may be warranted in such cases to reduce the risk of thrombosis.

E19a2 *BCR-ABL1 *is the most common variant transcript type in CML, yet molecular responses to TKI have rarely been evaluated, most likely to the unavailability of commercial plasmids necessary for construction of standard curves. While efforts to harmonise molecular methodologies that quantify e13a2 and e14a2 *BCR-ABL1* transcripts are underway [17], attainment of milestones of TKI therapy below complete cytogenetic remission such as major molecular remission [18] or complete molecular remission cannot be reproducibly assessed in such cases. Effective consensus or standardisation of these RQ-PCR assays, previously performed on only a case per case basis, is required.

This case serves to highlight the requirement for RQ-PCR monitoring, an essential component of modern disease management, for the less common *BCR-ABL1* transcript types during TKI dose reductions, interruptions, or changes in conjunction with conventional cytogenetic analysis.
